# Isolation and Characterization of Microsatellite Markers for *Mimusops balata* (Sapotaceae) and Cross-Amplification in Other *Mimusops* Species

**DOI:** 10.3390/plants1020100

**Published:** 2012-12-17

**Authors:** Stéphanie Dafreville, Cláudia Baider, F. B. Vincent Florens, Gérard Lebreton, Eric Rivière, Dominique Strasberg, Marie-Hélène Chevallier

**Affiliations:** 1 CIRAD, UMR C53 PVBMT, Saint-Pierre 97410, La Réunion, France; E-Mails: gerard.lebreton@cirad.fr (G.L.); riviere@cirad.fr (E.R.); marie-helene.chevallier@cirad.fr (M.-H.C.); 2 The Mauritius Herbarium, R. E. Vaughan Building, Agricultural Services, Ministry of Agro-Industry and Food Security, Réduit, Mauritius; E-Mail: clbaider@gmail.com; 3 Department of Biosciences, University of Mauritius, Réduit, Mauritius; E-Mail: v.florens@gmail.com; 4 Université de la Réunion, UMR C53 PVBMT, Saint-Denis 97715, La Réunion, France; E-Mail: umrc53@univ-reunion.fr

**Keywords:** conservation genetics, genetic diversity, La Réunion, Mauritius, nuclear microsatellites

## Abstract

*Mimusops balata* (Sapotaceae) is an endemic tree species from La Réunion and Mauritius. Like many species growing in lowland forests in La Réunion, it has suffered from human disturbances. We developed twelve microsatellite markers for *M. balata* and tested cross-amplification in five other *Mimusops* species to have powerful tools for genetic diversity studies. Genotyping peaks were of very low quality for two loci and were consequently abandoned for the genetic diversity analyses. Ten microsatellite loci were tested on 34 individuals of *M. balata* from two natural populations. The number of alleles per locus ranged from one to seven. The observed and expected heterozygosity levels varied from 0.000 to 0.823, and from 0.000 to 0.812 respectively. Two loci deviated from the Hardy-Weinberg equilibrium. The presence of null alleles was detected for one of these two loci. Nine to ten loci cross-amplified reliably in Mauritian species, for the other three species, four to six loci show successful amplifications. These polymorphic microsatellite markers are now available for population genetic investigations in *Mimusops* species aiming to establish accurate guidelines for conservation managers.

## 1. Introduction

La Réunion Island belongs to one of the thirty-four global biodiversity hotspots, where high concentration of endemism is undergoing severe loss of habitats [1]. Since the human colonization on the island, 70% of native vegetation have been destroyed or disturbed due to human activities [2]. Lowland forests have been particularly affected by anthropogenic disturbances like habitat destruction, forest fragmentation and logging. Preserving the last remnants of each habitat type on La Réunion is among the main conservation priority. To develop sustainable conservation plans, it is useful to assess information about the structure of genetic diversity and the patterns of gene flow between extant populations to reduce extinction risk [3].

*Mimusops balata* (Aubl.) C.F. Gaertn. (Sapotaceae) is one of the dominant endemic canopy trees that evolved in lowland forests of Mauritius and La Réunion. Like most of Sapotaceae species occurring on the Mascarene Islands, *M. balata* has an important keystone structure role in these forests. Its natural distribution has been severely contracted and fragmented. Although *M. balata* remained relatively abundant in its habitat [4], investigating its genetic diversity and differentiation are essential to ensure the long-term survival of this species and its functional role in its ecosystem. Here we report the development and characterization of twelve microsatellite markers for *M. balata* from La Réunion Island.

## 2. Results and Discussion

Twelve potential markers developed through 454 GS-FLX Titanium pyrosequencing were chosen to build a single primer mix for polymorphism analyses in the species *M. balata*. The characteristics of these twelve microsatellite loci are described in the Table 1. Genotyping peaks for the loci MbCIR26 and MbCIR15 were of low quality; allele assignment was therefore very difficult. So polymorphism analyses were not performed for these loci. The other ten markers were used to screen 34 individuals collected from two populations of *M. balata* (Les Makes and Sainte-Thérèse located respectively in the south-west and the south of La Réunion). The number of alleles per locus ranged from one to seven, with an average of 4.1 in Les Makes population and 4.5 in the Sainte-Thérèse population (Table 2). The observed and expected heterozygosities ranged from 0.000 to 0.823, and from 0.000 to 0.812 respectively. There was no evidence for linkage disequilibrium (*p* > 0.05) in any pair of loci. The locus MbCIR65 shows the lowest polymorphism levels. Two loci significantly deviated from Hardy-Weinberg equilibrium (MbCIR4, MbCIR30), suggesting the presence of null alleles, a Wahlund effect or an incomplete sample size. The presence of null alleles was confirmed at the locus MbCIR30.

**Table 1:** Characteristics of twelve microsatellite loci developed for the tree species *Mimusops balata*.

Locus	Repeat motif	Primer sequence (5' to 3')	Fluorescent Dye	Size range (bp)	Accession number
MbCIR4	(AC)_12_	F-TCTGTCATCCCAGTCTGCTG	VIC	89-113	JX413365
		R-TGTTTGATGTGTCTTTAGACCAAG			
MbCIR10	(GT)_13_	F-CCGTCTGCACCGTTCTTATT	6-FAM	132-154	JX413367
		R-TGATTTGCTTAAACTGGGAAGC			
MbCIR13	(CA)_14_	F-CAGGGATGGTAACAGCTGAC	NED	137-167	JX413368
		R-TGTTCCAATTACAGGTAAAGGC			
MbCIR14	(TG)_14_	F-TTTACATTAGATCGGTGCGAG	6-FAM	228-246	JX413375
		R-AGACCAAAATAAATACTGGGTATTACA			
MbCIR15	(CA)_14_	F-ATTATCCCATCAATAATCCCACC	NED	235-241	JX413376
		R-ATTCTTTTGTCTGTAGTTGTTTACTCA			
MbCIR26	(CA)_13_	F-AAATACATATAGTGCAACATAACCTGC	6-FAM	90	JX413374
		R-TGTTAGGTAGTGGTAGTCTCTGCTTT			
MbCIR30	(CA)_12_	F-GTGTTTTGCTTAAGGGTTGGTC	NED	72-94	JX413373
		R-CGAACGTGAATTACATGTGTTACC			
MbCIR34	(GA)_12_	F-AACTAACGGCCAAATAGGCA	PET	92-112	JX413366
		R-ATACCAGCAGAAAGCCAGCA			
MbCIR55	(TC)_12_	F-TTGCCTCCTGGAGTAAGTGG	PET	158-164	JX413370
		R-TCAGACAATTGGCCTTAGCC			
MbCIR62	(AG)_11_	F-GGACATGTTGCCAATGAGC	PET	230-252	JX413371
		R-GGGTATCATTAAACTTCTTGGTCA			
MbCIR63	(AG)_11_	F-CTTGCTGTGTAACTGGCAACA	VIC	234-246	JX413372
		R-TAGGTTCTCAGTCAGCAACATCT			
MbCIR65	(GA)_11_	F-CCAAGGGGCCTATTCTCTTC	VIC	149-161	JX413369
		R-CACCCAACGTACTCTGCTCA			

**Table 2:** Genetic diversity revealed by ten microsatellite loci in two natural populations of *Mimusops balata*. N Number of individuals tested; N_A_ Number of alleles detected; H_O_ Observed heterozygosity; H_E_ Expected heterozygosity; Significant deviation from Hardy-Weinberg equilibrium:* *p* < 0.05, *** *p* < 0.001.

Locus	Les Makes (N = 17)			Sainte-Thérèse (N = 17)		
	N_A_	H_O_	H_E_	N_A_	H_O_	H_E_
MbCIR4	7	0.625	0.812 *	7	0.588	0.708
MbCIR10	6	0.765	0.726	6	0.588	0.636
MbCIR13	6	0.588	0.710	6	0.471	0.456
MbCIR14	5	0.687	0.742	5	0.687	0.767
MbCIR30	4	0.294	0.614 *	3	0.000	0.581 ***
MbCIR34	4	0.588	0.533	7	0.823	0.700
MbCIR55	2	0.471	0.426	2	0.529	0.485
MbCIR62	3	0.625	0.523	5	0.823	0.706
MbCIR63	3	0.375	0.329	2	0.118	0.114
MbCIR65	1	0.000	0.000	2	0.062	0.062

The twelve loci were tested for cross-amplification in five other *Mimusops* species (Table 3): *M. petiolaris* (A.DC.) Dubard (MAU; N = 5), *M. erythroxylon* Bojer ex A.DC. (MAU; N = 5), *M. comorensis* Engl. (COM: Comoros; N = 3), *M. coriacea* (A.DC.) Miq. (MAD: Madagascar; N = 3), *M. elengi* L. (IN: India; N = 2). The genotyping peaks for MbCIR26 and MbCIR15 were of low quality too. Nine to ten loci successfully amplified in Mauritian *Mimusops* suggesting that these species are more closely related to *M. balata* than the other species tested in our study for which only four to six loci were successfully transferred. The number of alleles detected with these markers in the congeneric species ranged from one to eight.

**Table 3:** Cross-species amplification of ten microsatellite markers developed for *Mimusops balata* on species of the genus *Mimusops*. N: Number of individuals tested; N_A_: Number of alleles detected; MAU: Mauritius; COM: Comoros; MAD: Madagascar; IN: India.

Locus	N_A_ (Size range in bp)				
	*M. petiolaris*	*M. erythroxylon*	*M. comorensi* *s*	*M. coriacea*	*M. elengi*
	MAU (N = 5)	MAU (N = 5)	COM (N = 3)	MAD (N = 3)	IN (N = 2)
MbCIR4	7 (81–101)	5 (89–109)	4 (87–105)	2 (79–83)	1 (89)
MbCIR10	3 (132–136)	6 (126–150)	3 (136–162)	1 (138)	2 (140–156)
MbCIR13	5 (133–143)	6 (133–153)	3 (125–149)	-	-
MbCIR14	3 (228–240)	-	-	-	-
MbCIR30	5 (76–92)	3 (88–92)	-	-	-
MbCIR34	8 (98–136)	7 (98–136)	-	2 (112–114)	-
MbCIR55	2 (158–162)	4 (144–162)	1 (164)	1 (160)	1 (164)
MbCIR62	3 (246–250)	4 (246–252)	-	1 (234)	-
MbCIR63	4 (224–238)	3 (234–240)	2 (228–232)	2 (238–240)	2 (236–244)
MbCIR65	2 (155–161)	2 (155–161)	-	-	-

## 3. Experimental Section

DNA was extracted from dried leaf tissue of five individuals of *M. balata* using the DNeasy Plant Mini Kit (Qiagen) and sent to GenoScreen, France [5]. Microsatellite-enriched libraries were developed through 454 GS-FLX Titanium pyrosequencing by the platform GenoScreen (Lille, France) as described in [6]. It was a rapid and low-cost opportunity to develop a library of specific nuclear microsatellite markers for *M. balata*. This technique allowed the identification of 955 potential markers. Twelve dinucleotide microsatellite loci were chosen to build a single primer mix containing 2 µM of each primer (Table 1) for multiplex amplifications. Levels of variation of the twelve microsatellite loci were evaluated among 34 individuals from two populations of *M. balata*: Les Makes (N = 17) and Sainte-Thérèse (N = 17). Multiplex PCR was performed in 15 µL reaction volumes containing 10–20 ng of genomic DNA, 7.5 µL of 2× Type-it Multiplex PCR Master Mix (Qiagen), and 0.2 µM of each primer (in each primer pair the forward primer was fluorescently labeled, see Table 1). PCR reactions were carried out in a GeneAmp PCR System 9700 thermalcycler (Applied Biosystems) with the following conditions: 5 min of denaturation at 95 °C, 28 cycles consisting of 30 s of denaturation at 95 °C, 90 s of annealing at 60 °C, and 30 s of extension at 72 °C, and a final extension at 60 °C for 30 min. One microliter of PCR products was combined with 10.7 µL deionized formamide and genotyping was performed on an ABI PRISM 3130XL Genetic Analyser (Applied Biosystems), using 0.3 µL of Genescan AB500LIZ size standard (Applied Biosystems). Allele scoring was done using GENEMAPPER version 3.7 software (Applied Biosystems). The number of alleles, observed and expected heterozygosities and deviations from the Hardy-Weinberg equilibrium were calculated and tested with the software GENEPOP version 4.0 [7,8]. The linkage disequilibrium was tested using FSTAT version 2.9.3.2 [9,10] and the presence of null alleles was estimated by MICRO-CHECKER version 2.2.3 [11,12].

## 4. Conclusions

These results show the potential of these markers for investigating population genetic structure and diversity analyses throughout *M. balata* and closely related *Mimusops* species. Genetic diversity studies can generate helpful information for conservation managers that face the challenge of preserving biodiversity after severe human-induced disturbances.
